# Promoting health equity through health literacy interventions in educational institutions: a scoping review

**DOI:** 10.3389/fpubh.2026.1822447

**Published:** 2026-06-04

**Authors:** Belinda Agyapong, Vincent Israel Opoku Agyapong

**Affiliations:** 1 Department of Psychiatry, Faculty of Medicine, Dalhousie University, Halifax, NS, Canada; 2 Department of Psychiatry, Faculty of Medicine and Dentistry, University of Alberta, Edmonton, AB, Canada

**Keywords:** educational sector, health equity, health literacy, interventions, schools, scoping review, strategies

## Abstract

**Background:**

Disparities in health outcomes have been associated with inequitable access to health literacy and services. Additionally, reduced health literacy may lead to an unhealthy lifestyle, causing a decrease in quality of life and social inequities. Advancing health literacy is vital in educational organizations to curb gaps in social inequities. Adapting strategies to promote health literacy is essential for advancing equitable and effective educational organizations. Therefore, policies and interventions should aim to address any barriers that threaten inequities in the educational organization.

**Objective:**

This scoping review aims to explore how advancing health literacy strategies within the school organization can promote health equity.

**Methods:**

A scoping review of current literature was conducted using Arksey and O′Malley’s framework and reported using the PRISMA-ScR (Preferred Reporting Items for Systematic Reviews and Meta-Analyses extension for Scoping Reviews). Articles were identified using ERIC (Education Resources Information Center), PUBMED (Medical Literature Analysis and Retrieval System Online), EMBASE (Excerpta Medica Database), CINAHL Plus (Cumulative Index of Nursing and Allied Health Literature), and ProQuest Education Database. Included articles were restricted to the English language and the last twelve years to capture the most recent literature.

**Results:**

Thirty-seven interventions to advance health literacy in educational settings were identified, with most published between 2020 and 2025 (28/37). Twenty-nine targeted elementary to secondary school students, while fewer focused on teachers, counselors, school personnel, or university students. Most were individual-level interventions aimed at improving student health literacy, while organizational approaches emphasized staff development and whole-school practices. Strategies included peer-led programs, curriculum-embedded teacher-led interventions, digital tools, staff-focused training, and whole-school models. Mental health literacy predominated, though oral health, infectious disease, pain, emergency preparedness, and WaSH literacy were also addressed. All studies reported improved health literacy-related outcomes.

**Conclusion:**

Evidence indicates that educational institutions may advance health literacy through curriculum-based, digital, peer-led, and whole-organization strategies. Integrating these approaches into policies and practice may promote equity, improve student and staff outcomes, and position schools and universities as important settings for health promotion and future intervention research.

## 1 Introduction

Globally, health disparities remain a challenge, with inequities in health outcomes closely associated with social, economic, and educational factors. The global burden of disease remains substantial, with approximately 56 million deaths occurring annually, largely driven by avoidable or preventable non-communicable and communicable diseases (1). Global Burden of Disease estimates identify ischemic heart disease, stroke, and lower respiratory infections among the leading causes of mortality worldwide, while disability-adjusted life years (DALYs) continue to reflect a significant burden across younger populations (1, 2). The World Health Organization defines health as a state of complete physical, mental and social well-being and not merely the absence of disease or infirmity (3). Health has also been defined as the ability to adapt and to self-manage in the face of social, physical, and emotional challenges (4). In modern public health and resilience literature, “positive health” emphasizes resilience, meaningful functioning, social participation, and the capacity to manage life’s challenges (5). Worldwide, the disease burden for mental illness also indicates that the global burden of mental illness accounts for 32.4% of years lived with disability (YLDs) and 13.0% of DALYs (6). This cumulatively indicates an urgent need for improved health literacy. Published literature suggests a diverse definition of health literacy. Health literacy has been defined as individuals’ capacity to access, understand, critique, and utilize health information in healthcare, disease prevention, and health promotion (7). Obtaining, processing, understanding, and evaluating information is vital as it empowers an individual or group to act and make informed public health decisions that benefit the community (8). Organizational health literacy (OHL) is increasingly recognized as a systems-level construct that extends beyond individual competencies to the extent to which institutions enable people to access, understand, appraise, and use health information and services effectively (9, 10). Foundational work by Brach et al. identified ten attributes of health literate organizations, including leadership commitment, user-centered communication, workforce preparation, navigation support, and integration of health literacy into planning and evaluation processes (11). Subsequent scholarship has emphasized that OHL is central to reducing inequities because organizational demands often exceed the capacities of individuals, particularly those experiencing social disadvantage (12, 13). Although these frameworks were initially developed in healthcare settings, their principles are transferable to educational institutions, which similarly influence health through policies, communication systems, service pathways, and everyday environments. Schools, colleges, and universities can operationalize OHL through accessible health communications, inclusive curricula, staff training, supportive student services, and whole-school or whole-campus approaches that embed health into organizational culture (14, 15). Settings-based health promotion frameworks advanced by the World Health Organization further support this adaptation by recognizing schools and universities as critical environments for advancing health, wellbeing, and equity through systemic action rather than individual behavior change alone (16, 17). Accordingly, applying OHL concepts to educational settings offers a valuable framework for understanding how institutions can reduce barriers, strengthen health capabilities, and promote health equity across diverse learner populations. In addition, health literacy may be considered as an individual trait since individuals within the school environment, particularly students accessing, understanding, and utilizing health information, including mental health literacy, are embedded within, and influenced by, the broader organizational context (7, 18).

Health equity refers to the absence of avoidable, unfair, or remediable differences in health outcomes, ensuring all people have a fair opportunity to attain their full health potential (19). Such inequities are socially patterned and influenced by determinants including income, education, ethnicity, disability, geography, and language (20, 21). Limited health literacy often follows this social gradient and can reinforce existing disadvantage (8). It is associated with riskier health behaviors, poorer self-management, lower use of preventive services, worse overall health status, reduced quality of life, hospitalization, higher mortality, and increased healthcare costs (22–24). Conversely, when individuals can readily access, understand, and use health information, their capacity to make informed decisions and achieve better outcomes improves. Strengthening health literacy can therefore build individual and community resilience and contribute to improved health and wellbeing in increasingly complex societies (7).

Organizational health literacy provides an equity-oriented response by shifting responsibility from individuals alone to institutions that can reduce complexity, improve communication, and create inclusive environments responsive to diverse needs (12, 13). Educational institutions are particularly important settings because they engage diverse populations during formative life stages and influence wellbeing through policies, curricula, campus environments, and access to supports. By embedding equity-informed practices such as culturally responsive communication, accessible digital resources, targeted supports, and inclusive participation, schools, colleges, and universities can help reduce disparities and promote fair opportunities for health and academic success (14, 15).

Educational institutions are unique environments where health literacy may be fostered. The educational sector, including schools, colleges, and universities, plays a vital role in shaping the lives of individuals through their academic journey. The educational sector embodies the educators and offers a conducive and appropriate environment for the effective dissemination of messages on health and promotion of health literacy through the curriculum and lesson plans (25). Health literacy education may be embedded in the curriculum and implemented using a whole-school approach (26). An integrative review reported that poor social and socioeconomic conditions contribute to low health literacy levels, and educational attainment was a significant determinant of health literacy (24). Thus, greater health equity may be achievable by increasing health literacy within educational environments. It may also contribute to healthy lifestyle choices, decreased stress, and increased academic and occupational performance (24).

Policies and interventions that advance health literacy in educational settings are necessary to promote health literacy in schools. Additionally, increased health literacy in educational organizations can act as equitable access points for disadvantaged population, for health knowledge, preventive services, and supportive resources, thereby mitigating disparities that arise from broader social inequities (12). Despite increasing recognition of the importance of organizational health literacy (OHL), limited research has specifically focused on educational organizations, with comparatively fewer studies examining how health literacy principles can be adapted to schools, colleges, and universities (22, 24). Evidence regarding health literacy and its impact within educational organizations is also limited, with most studies focusing on the health sector, patients, and the health practitioner (27, 28). Arguably, understanding how educational organizations can systematically advance health literacy is vital, as this can contribute to health equity, which is essential for informing policy development, institutional leadership, and future research. In light of this, this scoping review aims to explore the scope of the existing evidence, key characteristics of health literacy initiatives in the educational sector, and their implications for health equity. In addition to the primary aim of the study, this review will also examine how advancing health literacy within educational organizations can contribute to promoting health equity. Furthermore, the review will identify existing gaps in health literacy strategies in the educational sector and identify opportunities for future research.

## 2 Methods

### 2.1 Study design

This scoping review was conducted in adherence to the Preferred Reporting Items for Systematic Reviews and Meta-Analyses Extension for Scoping Reviews (PRISMA-ScR) statement (29). A comprehensive search strategy that allows replicability, reliability, and transparency was adopted. The review also followed Arksey and O’Malley’s five-stage approach to scoping reviews (30). This involves the following steps: developing the research question, searching for relevant studies, article selection, charting the data, data extraction and collating, summarizing, and reporting the results.

### 2.2 Developing the research question

The research question was: “What are existing strategies or interventions that promotes health literacy in the educational sector?

### 2.3 Identifying relevant studies

A systematic literature search was conducted in the following databases: ERIC (Education Resources Information Center), CINAHL Plus with Full Text (Cumulative Index of Nursing and Allied Health Literature) via (EBSCOhost interface), PubMed (Public/Publisher MEDLINE) (NLM journal articles database), EMBASE (Excerpta Medica Database), and ProQuest Education database. The included articles were restricted by publication year (2013–2025). This cut-off year 2013 to 2025 was selected to capture the most recent and relevant body of literature on health literacy strategies and interventions, while maintaining a sufficiently broad time span to identify key foundational studies, developments and trends. The search consisted of keywords representing concepts of strategies or interventions focusing on health literacy to promote equity in educational organizations. The specific MeSH terms, keywords, and descriptors are listed below.

#### 2.3.1 Sample search strings-PubMed

(“health literacy”[Title/Abstract] OR “health literacy strategy”[Title/Abstract] OR “health literacy strategies”[Title/Abstract] OR ((“health literacy”[MeSH Terms] OR (“health”[All Fields] AND “Literacy”[All Fields]) OR “health literacy”[All Fields]) AND “policy”[Title/Abstract]) OR “health literacy policies”[Title/Abstract] OR “health literacy techniques”[Title/Abstract] OR “health literacy interventions”[Title/Abstract]) AND (“education”[Title/Abstract] OR “school”[Title/Abstract] OR “school environment”[Title/Abstract] OR “education system”[Title/Abstract] OR “educational organization”[Title/Abstract] OR “educational sector”[Title/Abstract] OR ((“educability”[All Fields] OR “educable”[All Fields] OR “educates”[All Fields] OR “education”[MeSH Subheading] OR “education”[All Fields] OR “educational status”[MeSH Terms] OR (“educational”[All Fields] AND “status”[All Fields]) OR “educational status”[All Fields] OR “education”[MeSH Terms] OR “education s”[All Fields] OR “educational”[All Fields] OR “educative”[All Fields] OR “educator”[All Fields] OR “educator s”[All Fields] OR “educators”[All Fields] OR “teaching”[MeSH Terms] OR “teaching”[All Fields] OR “educate”[All Fields] OR “educated”[All Fields] OR “educating”[All Fields] OR “educations”[All Fields]) AND “segment”[Title/Abstract])) AND (((“reduce”[All Fields] OR “reduced”[All Fields] OR “reduces”[All Fields] OR “reducing”[All Fields]) AND “inequities” [Title/Abstract]) OR ((“limit”[All Fields] OR “limitation”[All Fields] OR “limitations”[All Fields] OR “limited”[All Fields] OR “limiting”[All Fields] OR “limits”[All Fields]) AND “inequities”[Title/Abstract]) OR ((“avoid”[All Fields] OR “avoidability”[All Fields] OR “avoidable”[All Fields] OR “avoidance”[All Fields] OR “avoidances”[All Fields] OR “avoidant”[All Fields] OR “avoidants”[All Fields] OR “avoided”[All Fields] OR “avoider”[All Fields] OR “avoiders”[All Fields] OR “avoiding”[All Fields] OR “avoids”[All Fields]) AND “inequities”[Title/Abstract]) OR “decrease inequities”[Title/Abstract])

### 2.4 Articles selection

Two independent researchers reviewed the citations during the title and abstract screening and full-text review phases based on specific eligibility criteria. All discrepancies at the title and abstract screening or full-text review stages were resolved through discussion and consensus. Meta-analyses, systematic reviews, case reports, graduate student theses, opinion pieces, commentaries, editorials, or grey literature like non-peer-reviewed articles, non-research articles, and conference reports were excluded. Articles were limited to original, peer-reviewed articles written in English. Articles were also excluded if the main focus was not on health literacy or on the educational sector.

### 2.5 Data extraction and charting

A standardized data extraction form was developed by researchers and used to chart key study characteristics, including author, year of publication, country, study design, sample size, measurement tools, health literacy strategies/Intervention, and main findings. The extracted data were summarized into a table and validated by a second reviewer to ensure accuracy.

## 3 Results

### 3.1 Overview of included studies

**Figure 1:**
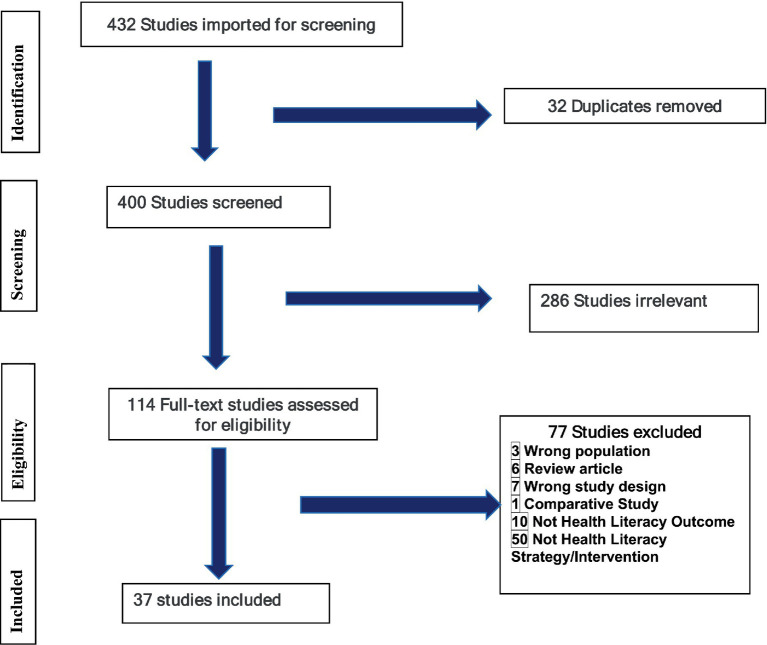
PRISMA flow diagram outlining the selection process.

**Figure 2:**
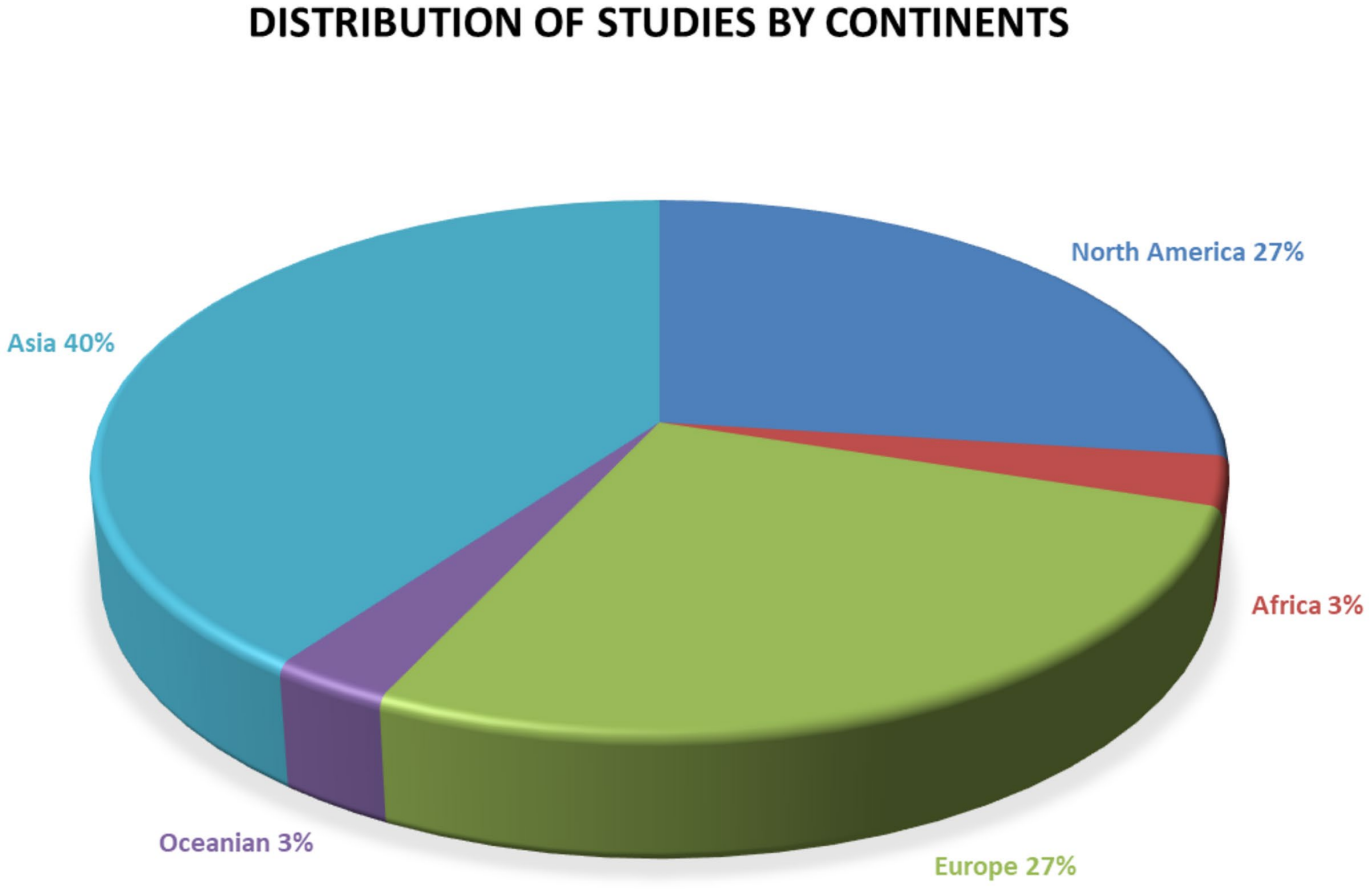
Distribution of studies by continents.

A total of 432 citations were imported into Covidence software (31), and 32 duplicates were identified and automatically removed. Of the 400 studies that remained for abstract screening, 286 studies were excluded since they did not meet the eligibility criteria, and 114 studies were screened at the full test stage. Of these, 77 studies were excluded, and 37 studies were included in this scoping review, as illustrated in Figure 1. Across these 37 studies, a total of 20,092 participants, the majority of whom were students. The sample sizes in the included articles (Tables 1, 2) ranged from 12 to 4,600. The ages of the students ranged from 5 to 21 years, while for the other participants, the age spanned from 19 to >60. The studies were published between 2013 and 2025. Figure 2 provides the distribution of studies by continents. Most studies were conducted in Asia (40%), followed by Europe (27%), North America (27%), Africa (3%), and Oceania (3%). Notably, no studies were conducted in South America, as illustrated in Figure 2.

**Table 1:** Interventions to advance health literacy in the educational sector.

Author/year/country	Study design	Measurement scales	Educational sector/population	Age range	Sample size	Health literacy program/intervention	Duration	Findings/outcome
Adefunke, 2025 (71), United States (US)	Randomized controlled trial design	The Child and Youth Resilience Measure (CYRM-R) is a 7-item assessment of resilience. Self-Efficacy Questionnaire for Children The Patient-Reported Outcome Measurement Information System (PROMIS) Emotional Distress Anxiety (8-item) and Depressive Symptoms Scales (8-item)	5–6th Grade Students	11–13 years	109	Advocates 4 ALL Youth (ALLY) A universal, 6-week mental health literacy program	6-weeks	58% youth in the treatment group endorsed negative affect at baseline, and this was reduced to 53% post-program delivery. Youth in the treatment group had a significantly greater improvement in emotional self-efficacy and psychological stress.
Akca, 2021 (37) Turkey	Randomized controlled trial	Turkey’s Health Literacy Scale (THLS-32)	Nursing students	Mean-19.97(SD-1.84)	206	Health literacy education	12 weeks	The health literacy level of the intervention group was significantly higher than that of the control group.
Bjørnsen, 2018 (69), Norway	Sample cohort data collection T1 and T2	Positive MHL was measured by the 10-item Mental Health Promoting Knowledge (MHPK-10) scale. Anxiety and depression were assessed using the 10-item Hopkins Symptom Checklist (HSCL-10)	Upper secondary schools	15–21 years	357	MEST: working strategy for school health to positive MH and mental well-being	2016/2017 school year.	Positive MHL increased significantly more among the MEST participants (69)
Booth, 2023 (45), Dublin, Ireland	Comparative design	Author-designed questions about mental health knowledge, 10-item version of the General Help-Seeking Questionnaire	Secondary school students	12–16 years	536	Peer-led initiatives	Two to four weeks	Students’ mental health knowledge and help-seeking intentions significantly improved in both peer- and adult-led groups.
Curtin, 2024 (46), England	Purposive recruitment, qualitative, observation	Observations during intervention delivery, focus groups, and interviews shortly after the programme.	Secondary school students	Peer educators 14–18 years peer learners −11–13 years	134	Peer Education Project	2020 to 2022	The programme engendered a shift in school culture across the population that influences psychological and physical well-being. Through this cultural shift, stigma around speaking about mental health dissipated, leading to a school culture that prioritizes mental health support. The PEP promoted an increased awareness of existing sources of support among students and staff and often led to staff making tangible changes to the support infrastructure, aligning with the whole-school approach.
Elyamani, 2024 (32), Qatar	Randomized control trial.	Self-administered tool developed by the WHO to be used in the EMRO region as part of the implementation plan for the WHO EMRO School Mental Health Package.	secondary schools’ teachers	Average age was 41.5 (SD 7.2) years	195 teachers	World Health Organization School Mental Health Program (WHO-SMHP)	3 months	Compared with controls, teachers from the intervention group demonstrated a significant improvement in the level of MHL at day three and after three months
Habgood, 2025 (35), Victoria, Australia	Pre-post cohort study design	Survey, focus groups/interviews	Teachers and students	5–8 years	228 Teachers and students	The Decode Mental Health and Wellbeing Program (Decode)	4-weeks	Decode was acceptable to both teachers and students, with strong endorsement of the program’s relatability, engagement, and appropriateness. The program led to improvements in student and teacher mental health literacy, including knowledge of help-seeking strategies, reductions in stigma, and improved teacher-observed student mental health and wellbeing.
Hosseini, 2025 (43), Gorgan, Iran	Randomized controlled field trial	Valid and reliable Test of Oral Health Literacy in Adults (TOHLA)	Four public secondary school students	15–18 years	140 female	A school-based intervention in enhancing oral health literacy	2 Months 45-min sessions	The intervention significantly improved the total oral health literacy score, as well as its cognitive, behavioral, media, and communicational domains in the experimental group
Hudson-Rose, 2023 (47), United States	Pretest to post-test implementation	A 10-question pretest and an identical 10-question posttest.	Middle and high school students	NR	223 students	A three-part cancer education curriculum	Lessons 1, 2, and 3; 30–40-min cancer-related educational intervention	The results demonstrate that the three-part cancer education curriculum intervention can significantly increase Appalachian Kentucky middle and high school students’ cancer literacy.
Hughes, 2018 (40), US	Pre- and posttest design	17 content questions, 8 additional feedback questions. Teacher reflections and student pre- and posttests to measure knowledge transfer gained and gather feedback	Students	NR	948 students	The revised 4-module version of the Navigating the Health Care System unit	Two 90-min classes/Four, 45-min modules over the course of 2 days in each classroom in the 2014–2015 school year	Student knowledge increased from pretest (64%) to posttest (82%). Students reported increased health literacy.
Jacque, 2016 (44), US	A quasi-experimental design	pre- and post-conceptual knowledge inventory	Four public high schools plus a private school in 11th and 12th grades	NR	398	An innovative high school biology curriculum focused on infectious disease (ID) in Biology II classes. The ID module is built around five questions	2010 and 2013	HL skills in seeking, interpreting, and evaluating current health-related information. The results presented here demonstrate that our health-contextualized biology curriculum was effective in developing these health literacy -relevant skills. Participants in each school setting demonstrated increases in conceptual content knowledge as well as in understanding how to apply scientific principles to health claims evaluation and risk assessment, and in self-efficacy toward learning about ID.
Khanal, 2025 (75), Nepal	Pre-test post-test quasi-experimental design	Standardized self-administered questionnaire- health literacy awareness, (3) General Self-Efficacy Scale (GSES), (4) health literacy S-Child-Q24-NEP, and (5) Adolescent Health Promoting Short Form Scale	Community secondary school students	13–19 years	468	A school-based health literacy intervention -Followed the basic steps of the intervention mapping approach	6 Months	The intervention significantly improved health literacy awareness, self-efficacy, and overall health literacy, including four dimensions (access, understanding, appraising, and applying health information) across three health domains (health care, disease prevention, and health promotion).
Kilgour, 2015 (58), UK	Practice-based, qualitative study	Six focus group semi-structured interviews were conducted using nine open-ended questions exploring the perception of the meaning of health literacy	Year 9 and Year 11	13–16 years,	34 pupils	Delivering key health messages through the taught curriculum and other on-site provision.	Focus group interviews lasted between 50 and 80 min, and individual interviews lasted between 45 and 60 min.	Findings suggest that pupils and staff have an understanding of health and a capacity for health literacy, through health education (via taught subjects), which is not statutory across the four Key Stages of the National Curriculum.
Konig, 2022 (74), Germany	Pre-post measurement study	10-item instrument. The Health Literacy for School-Age Children Instrument.	10th grade, Higher grade, vocational schools, school-age children	Average age of 17.88 (SD 1.22) years	323	Newly developed e-learning course	7 days	Participants showed higher health literacy levels Higher competency levels in the domains of theoretical knowledge, practical knowledge, critical thinking, self-awareness, and citizenship
Kutcher, 2017 (41), Tanzania	Survey	Questionnaire- 36-item Mental Health Knowledge and Attitude Test developed to accompany The Guide. School Mental Health Literacy Impact Data Collection Form	Students	NR	4,600 were taught MHL	African Guide (AG), a classroom-ready curriculum resource addressing all aspects of mental health literacy	one year	Students demonstrated improved or very much improved knowledge, attitudes, and help-seeking efficacy, with similar outcomes reported for teachers. Results of this study demonstrate a substantial positive impact on MHL-related activities and outcomes for both students and teachers using the AG resource in Tanzania.
Mokmin, 2021 (38), Malaysia	Both quantitative and qualitative methods, a semi-structured interview, and analytic sessions	The users’ acceptance of the technology was measured using the Unified Theory of Acceptance and Use of Technology 2 (UTAUT2).	Undergraduate students	20 to 23 years	75	A chatbot developed to educate users and provide health literacy	two months	Chatbots have significant potential and can be utilized as conversational agents to increase health literacy, especially among students and young adults.
Montañez, 2023 (35), New York City	A pre−/post quasi-experimental design	A survey adapted from validated measures of MH knowledge and attitudes	Elementary school personnel and students.	20- > 60	109	Turn2Us is an evidence-based MHL intervention	20-weeks	Improvements in MHL of school staff, which may facilitate staff’s ability to identify and support students’ MH needs

MHL, mental health literacy; MEST, short version of the Norwegian word for coping is a school-based MHL working strategy for school health services; WHO-EMRO, World Health Organization, Eastern Mediterranean Region; SMHP, School Mental Health Program; WHO, World Health Organization.

**Table 2:** Interventions to advance health literacy in the educational sector.

Author/year/country	Study design	Measurement scales	Educational sector/population	Age range	Sample size	Health literacy program	Duration	Findings/outcome
Nguyen, 2020 (34), Vietnam, Cambodia	Randomized trial pre-post randomized design	The Vietnamese version of the Mental Health Literacy Scale The 21-item Beliefs Toward Mental Illness Scale-BMI 36-item Mental Health Knowledge and Attitude Test developed to accompany The Guide	Teachers and students in 6th to 12th grade	Students-median age: 15. Teachers- median age of 36	80 teachers and 2,539 students from 20 schools in Vietnam (Study 1), and 67 teachers and 275 students in one school in Cambodia (Study 2). Total = 2,961	Mental Health & High School Curriculum Guide	3-day teacher training, followed by delivery of the 6-module classroom-based MHL curriculum to their students over five weeks. Staff 2-day training, Students- delivered over six weekly sessions (approximately 1 to 1.5 h per week)	With limited adaptation, a teacher-delivered MHL intervention can produce measurable increases in MHL among teachers and students. Students who received the Guide-VN program showed significantly higher levels of mental health knowledge. For the teachers, all but one variable MHL Stigma showed significant within-group improvement in the intervention group. MHL Stigma Recognition scores for teachers in the control group significantly worsened from T1 to T2
Ojio, 2019 (48), Japan	Pre, post, and follow-up	Questionnaire about knowledge about mental health/illnesses, Recognition of the mental health state of a character in a vignette	Grade 5 to 6 students from nine Japanese elementary schools	Grade 5 to 6 mean age (SD) = 10.6 (0.5)/11.7 (0.5),	631	A concise teacher-led program for mental health literacy (MHL). The program consisted of a 45-min session, delivered by school teachers using a 10-min animated film	45-min session, delivered by school teachers using a 10-min animated film. Post text. 3 months	Significant improvement in knowledge about mental health/illnesses, recognition of mental health state, and intention to help peers/seek help when suffering from mental health problems, were significantly improved immediately and 3 months post-intervention.
Ojio, 2015 (79), Japan	Pre- and post-tests and pre- and follow-up tests questions vignette Questionnaire	A self-report questionnaire	grade-9 secondary students	14–15 years	118	School-based mental health literacy education program.	Once a week, over 2 weeks, two 50-min sessions for 3 months	Knowledge of mental health/illnesses and desirable behavior for help-seeking were significantly improved immediately after (post-test). Intentions to seek help and to help peers with mental health problems were also significantly (*p* < 0.001) elevated at post-test and at 3 months compared with the pre-test.
Papa, 2023 (39), Italy	Quantitative and qualitative	A pre-post-test questionnaire was developed and used to collect students’ and educators’ feedback.	Students of health-related degrees	19 and 42 years	107	Pan-European health literacy educational programme	Pilots Test 1–4 November 2018 to October 2019	The pre-post test showed a significant improvement in health literacy awareness after the training.
Patchasuwan, 2022 (42), Thailand	Participatory action research mixed methods	Questionnaire developed by the researchers	School administrators, teachers, senior high school students, health officers, and health volunteers	NR	60–3 school administrators, 5 teachers, 45 senior high school students, 2 health officers, and 5 health volunteers	A school-based health literacy model for liver fluke prevention and control	once a week 3 months	The participants’ health literacy and practical skill mean scores increased after the intervention.
Sangalang, 2022 (56), Philippines	Cluster randomized controlled trial	Questionnaires to measure health literacy	Grades 5, 6, 7, and 10, from 15 schools	<13 years	756	Health education (HE), WaSH policy workshops, provision of hygiene supplies, and WaSH facilities repairs	June 2017 to March 2018	After the intervention, the proportions of children who received a “passing” score (≥60%) for overall health literacy, overall knowledge about germs, and overall knowledge about handwashing increased across all study arms.
Simkiss, 2023 (80), UK	A randomised controlled trial	Knowledge and Attitudes to Mental Health Scales (KAMHS)	Year 9 pupils	Aged 13–14	1,926	Cymru mental health literacy intervention programme	12-week intervention programme	Significant improvement in all aspects of mental health literacy, including mental health-related behaviors and help-seeking behaviors, with a reduction in stigmatic beliefs about people with mental health problems, was achieved after completing the Guide Cymru programme.
Skre, 2013 (76), Norway	A non-randomized cluster-controlled trial included adolescents	A 66-item questionnaire, of which 7 questions were open-ended	Secondary schools	13–15 years	1,070	mental health literacy, a universal education programme	three-day intervention	Mental health literacy improved after the intervention, and there was a shift toward suggesting primary healthcare as a place to seek help.
Soon Ken, 2024 (36), Malaysia	One group pre-test/post-test design	Counseling Self-Efficacy scale (COSE), Mental Health Literacy Scale (MHLS), and Self-Compassionate Scale – Short Form (SCS-SF).	School counselors	NR	28	Training module named MDAS (Mental Health Disorder, Developmental Disorders, Attachment and Self-Compassion) program	4-month	Significant increment of COSE and MHLS scores after MDAS training
Suwono, 2023 (25), Indonesia	Quasi-experimental with a non-equivalent pre-test – post-test control group design.	Biology-related health test (BRHT), health literacy test (HLT), and PBL Perception Scale.	Four classes, consisting of tenth-graders,	NR	122	Problem-based learning (PBL) of biology through the biology curriculum. Lessons in biology are chosen as a platform for health literacy development	16 weeks.	Improvement in biological knowledge and health literacy. Improved students’ biological knowledge in health and literacy, better than traditional methods.
Valerie, 2025 (53), Ohio US	Pretest and posttest	The pretests and posttests for each chapter responses for knowledge, attitudes, and behaviors	Third graders in elementary school in the southwest	Aged 8 to 10 years old	246	A digital curriculum called the eBook for Oral Health Literacy	An eight-week program that included approximately 25 min of the oral health literacy intervention during each class.	The intervention showed promise in changing oral health knowledge and behaviors among third graders.
Wager, 2018 (49), Germany	Pre-post design	A knowledge questionnaire designed by the authors. The Pain Knowledge Questionnaire for Children (PKQ-CH). This final version consists of 20 single-choice items. The questionnaire was designed so that each section of the movie was represented by at least one question.	5th to 7th grade	10 to 15 years, with a mean age of 11.7 years	95	School-based health educational movie programs	11-min educational movie. The movie format was chosen to be self-explanatory	There was a significant increase in pain knowledge for all participants,
Wang, 2023 (55), China	Randomized controlled trial	The Adolescent Health Literacy Evaluation Scale under Public Health Emergencies (AHLES-PHE), the Depression Self-Rating Scale for Children (DSRSC), the Generalized Anxiety Disorder 7-item scale (GAD-7), and the Simplified Coping Style Questionnaire (SCSQ).	Grade 7 and Grade 8 junior middle schools	13.1 ± 0.7	724 students	Health education courses related to public health emergencies: 12 classes, one per week.	March to December 2022	After the courses were completed, the AHLES scores of the course group improved compared with those before the course, and the difference was statistically significant.
Weng, 2025 (52), Taiwan	Quasi-experimental, pre-post design	Structured, validated instruments questionnaire. Health literacy was evaluated using a 17-question instrument	Senior high school students	16 and 18 years	768	The interactive digital intervention (IDI) program includes 6 units, each incorporating interactive e-books complemented by responsive PowerPoint presentations (Microsoft Corp), learning worksheets, animated videos, and game-based modules.	NR Includes two 50-min sessions on substance use prevention	The IDI had a significant positive impact on students’ substance use prevention outcomes. An intragroup comparison revealed that the IDI group had significantly greater improvements in knowledge, health literacy, functional literacy, critical literacy, communicative literacy, and learner engagement.
Yamaguchi, 2024 (54), Japan	Cluster randomized controlled trial	A self-report questionnaire pre- and post-intervention	grade 10 students	Age 15–16	270	Online MHL education, which included an animated video	20-min session	All outcomes were significantly improved in the intervention group compared to the control group post-intervention, except for “intention to seek help.”
Yamazaki, 2024 (81), US	Case study pre- and post-project assessment data	Pre- and post-project scores from the Health Literacy Assessment Scale for Adolescents	Students	NR	12	All of Us data consisted of two broad categories of activities: education and research.	January and June 2023	The project was successful and met its goal of increasing students’ health literacy. The project provided a return on value by increasing their health literacy and research skills, which, in turn, helped raise the community’s awareness of health issues
Yifeng, 2023 (60), Canada	Naturalistic cohort study, pre- and post-test	30-item knowledge items addressing basic understanding of the brain and common mental disorders	Grade 9 students	(ages 14–15)	332	The Guide, evidence-based MHL curriculum resources	One year	Students showed significant short-term and long-term improvements in knowledge and reductions in stigma. Educators also showed substantial short-term improvements in knowledge and reductions in stigma.
Yifeng, 2025 (51), Canada	Longitudinal cohort study	Validated measures to assess mental health knowledge, stigma, help-seeking attitudes, validated World Health Organization Wellbeing Index, and mental health 30 knowledge-related questions	Grade 8–9 students. Secondary students	Ages 13 to 15 years	523	The Guide, standardized MHL interventions	Require approximately eight to 12 h of in-person classroom time and are delivered by teachers. Beginning in grade 8 and ending in grade 9	Improvements in knowledge and stigma at all measurement intervals, and statistically significant differences in help-seeking and well-being outcomes were not observed Improved and sustained students’ mental health knowledge while reducing stigma about mental illness.
Zare, 2021 (50), Iran	Pretest–posttest and control group design. Cluster multi-stage sampling	Adolescent MHL questionnaire	Eighth-grade female students. High school students	(13–15 years)	220	MHL program. The Mental Health and High School Curriculum Guide, version three	Six 60- to 90-min sessions	After completing the training programme, the intervention group showed a significant improvement in MHL.
Zaza, 2023 (26), Canada	Pre-post study Online survey	The 10-item International Positive and Negative Affect Schedule (Short Form; IPANAS-SF) The 12-item Mental Health Knowledge Schedule (MAKS)	Undergraduate	Mean age was 21.4	40	Undergraduate course on mental health literacy	12- week, 0.5 credit course	Students made significant gains from T1 to T2, with a large effect size, in terms of attitudes toward seeking mental health services.

### 3.2 Interventions to advance health literacy in the educational sector

Thirty-seven interventions aimed at advancing HL in the educational sector were identified, as shown in Tables 1, 2. Nine studies were from 2013 to 2019, and twenty-eight from 2020 to 2025.

### 3.3 School levels

Twenty-nine studies addressed the health literacy of students from elementary to secondary school or high school. Only one study specifically focused on secondary school teachers (32). Two studies focused on both teachers and students (33, 34), and one study focused on elementary school personnel and students (35), and one on school counselors (36). Four studies reported on intervention with emphasis on undergraduate students, nursing students, and students of health-related degrees (26, 37–39).

### 3.4 Organizational levels

The majority of the interventions were individual-level, primarily targeting students’ health literacy skills. Organizational interventions most commonly focused on staff professional development, and school-wide engagement in health literacy practices, and policy consistent with whole-school approaches to health promotion (32–35, 40–42). Other strategies were deployed across multiple public high schools within the jurisdiction (43, 44).

### 3.5 Target-groups health literacy interventions

A broad range of health literacy strategies was identified. These can be categorized as peer-led interventions, curriculum-embedded interventions delivered by school teachers, digital HL strategies, staff-oriented interventions, and whole-school approaches. Peer-led approaches (45, 46) led to a shift in school culture across the population and positively influenced the psychological and physical well-being of students. Curriculum-embedded interventions, which include subject-based approaches, are teacher-led interventions including the mental health and high School Curriculum Guide, cancer education curriculum, animated teacher-led sessions and educational movie (47–50). Other curriculum-embedded interventions include the Guide, a standardized mental health literacy (MHL) intervention (51) originally utilized in Tanzania (41), and the Mental Health & High School Curriculum Guide (34). Subject-based classroom strategy, normally centered on biology as a subject, and includes an innovative high school biology curriculum or problem-based learning of biology (25, 44).

### 3.6 Digital tools

Digital health literacy for instance includes the online mental health literacy or the interactive digital intervention program (52), a digital oral health eBook (53), which demonstrated positive changes in knowledge and behavior among elementary students and an online MHL module or a chatbot (38, 54).

### 3.7 Topics of health literacy

MHL particularly was dominant among all the health literacy strategies, however, other studies, broaden the conceptualization of health literacy in schools to include, oral health literacy (53), pain knowledge (49), public health emergency preparedness (55) and water, sanitation, and hygiene (WaSH) literacy (56). The findings from this scoping review show that all the identified health literacy strategies implemented in the school setting resulted in some improvement in the health literacy of students, staff/teachers, and school counselors.

## 4 Discussions

This scoping review mapped the current literature on strategies to advance health literacy (HL) within educational settings and examined their implications for health equity. Diverse strategies were implemented across elementary, secondary/high schools, and tertiary education. Overall, the findings suggest that advancing HL within educational organizations is associated with enhanced knowledge, increased self-efficacy, increased help-seeking intentions, reduced stigma, and improvements in well-being.

### 4.1 School health literacy

Effective implementation of health literacy training and practices within the school community requires sustained professional development alongside the systematic integration of health literacy into routine practices through a whole-school approach (57). This process also necessitates the adoption of structured and effective communication strategies, both written and oral, to ensure accessibility, thereby strengthening health literacy across the school environment.

A whole school health literacy approach may be adopted at three levels: ‘micro’, ‘meso’, and ‘macro’, all of which may offer substantial benefits. The ‘micro’ level may involve embedding health literacy into everyday classroom practices and teacher-student interactions (25, 44, 58). At the ‘meso’ level, schools and organizations may align leadership, policies, and professional learning to support consistent implementation, indicating greater organizational commitment to health literacy (59). Finally, at the ‘macro’ level, supportive policy frameworks and system-wide priorities are required to enable and sustain health literacy initiatives across the education sector. Health literacy programs such as the World Health Organization School Mental Health Program (WHO-SMHP) may be adapted throughout the educational system (32). Research suggests that health literacy interventions in schools maybe more effective and sustained when embedded within the curriculum (25, 34, 51, 60) rather than delivered as one-time or short-term activities. In particular, curricular integration enables HL development to move beyond individual-level learning (micro) and become embedded within school structures and teaching practices (meso), while also supporting broader policy and system-level approaches to health promotion (macro). This aligns with evidence emphasizing that whole-school and community-linked approaches are more likely to create lasting improvements in health literacy and equity (61) Therefore, incorporating HL into ongoing curricular activities may strengthen both the sustainability and broader systemic impact of school-based interventions. Leadership and organizational commitment are also vital in helping workers improve their interpersonal communication, contributing to increased accessibility to health education materials (59).

### 4.2 Advancing health literacy in the educational settings

Within the educational context, organizational HL may include curriculum design, school culture, staff capacity, communication systems, and partnerships that collectively shape students’ and educators’ health-related behaviors and outcomes. The findings of this scoping review are fundamental to reducing inequities that originate from differential access to health information and support. Most countries have compulsory education policies, particularly for elementary school students (62–66), presenting a unique opportunity to advance health literacy in schools as all students are reached regardless of their socioeconomic background. Advancing health literacy within educational settings may be an important pathway to promoting health equity. Health inequities are socially produced, avoidable, and closely linked to social determinants such as education, income, language, and social exclusion (20, 21, 67). Because limited health literacy disproportionately affects socially and economically marginalized populations, schools, colleges, and universities are well-positioned to reduce disparities by creating environments in which learners can access, understand, and use health information effectively (8, 12).

Health information can be integrated and disseminated through the curricula and other school-based practices, thereby supporting the promotion of health equity; such approaches should therefore be encouraged and strengthened (58). When students are well-informed, they will make informed health and social choices, which will ultimately benefit society. For instance, these benefits have been reflected in improvements in knowledge, recognition of mental health conditions, and help-seeking behaviors (34, 41, 44). With the increased global burden of mental health conditions (6), health literacy is vital as early detection is essential for diagnosis and prognosis. Advancing health literacy is noted to help redesign systems that maximize individuals’ capacities to learn how to maintain good health, manage illness, communicate effectively, and make informed decisions, which promotes equity (11). The outcomes of this scoping review demonstrate that educational institutions can serve as effective platforms for embedding health literacy principles. For instance, whole-school and peer-led strategies (45, 46) led to shifts in school culture, increased mental health awareness, and improved help-seeking behaviors. The whole-school approach has been reported to offer students the best opportunities to achieve a critical understanding of health literacy (61). The childhood and adolescence stages are critically vital phases of life where young people develop autonomy, self-control, social interaction, and learning, making this period crucial for health literacy (68). Additionally, health literacy gains during this stage directly influence their health outcomes for the rest of their lives. Advancing health literacy within the educational center aligns with the Health Promoting Schools framework of the World Health Organization (WHO) (68), which emphasizes systemic integration of health literacy into school policy, curriculum, and community engagement. Importantly, interventions such as MEST and the WHO School Mental Health Program (WHO-SMHP) significantly advanced positive mental health literacy (MHL) among participants, illustrating that when schools consistently support health literacy implementation and staff engagement, gains in MHL are more sustainable (32, 69). Findings from this scoping review also reveal a notable increase in research activities on health literacy strategies in the educational sector over the last five years (2020–2025), compared to earlier periods. This may indicate a heightened awareness of the role schools play as educational institutions in shaping the health outcomes of the students.

Many studies in this review focused on improving students’ knowledge, confidence, and practical skills, reflecting functional and interactive approaches to health literacy that may positively influence both educational and health outcomes. Examples included improved cancer literacy through curriculum-based education (47), enhanced ability to navigate healthcare systems (40), and gains in oral health literacy and hygiene practices (43). Such structured, curriculum-integrated interventions may help alter life-course trajectories for learners at greater risk of adverse outcomes and reduce inequities among disproportionately affected groups.

Importantly, equity is advanced not only through universal interventions but also through proportionate and inclusive strategies that respond to differing levels of need. Educational organizations that provide culturally responsive communication, accessible digital resources, targeted supports, and meaningful opportunities for participation may be especially effective in addressing barriers faced by underserved populations (13, 15). This aligns with models that position health literacy as both a determinant and an outcome of health inequities (7). Accordingly, embedding health literacy within institutional policies, curricula, and support systems may enable educational institutions to function as equity-promoting organizations that foster fair opportunities for both health and academic success.

Teacher-focused interventions (32, 35) further highlight the importance of the school organizational dimension of health literacy. When staff MHL improves, they are better positioned to identify the needs of students early, reinforcing the fundamental role of educators in advancing health literacy and reducing inequities. These findings are consistent with evidence suggesting that improving provider or institutional health literacy responsiveness can mitigate disparities in service utilization and outcomes and healthcare costs (70).

### 4.3 Mental health literacy

Notably, a substantial proportion of included studies focused on MHL (26, 33, 41, 45, 51, 71) with reported improvements in mental health knowledge, emotional self-efficacy, stigma reduction, and help-seeking efficacy. Given the rising global burden of youth mental health conditions (72), prioritizing MHL in schools is timely and aligns with global mental health promotion strategies. However, the dominance of MHL interventions also highlights a relative paucity of research addressing other dimensions of health literacy, such as digital health literacy. As digital health platforms become increasingly important sources of mental health information, the growing spread of misinformation highlights the need to address poor literacy and communication skills by integrating digital literacy competencies into school curricula to support and strengthen MHL (18, 73).

### 4.4 Intervention duration

There was heterogeneity in intervention duration and delivery modes, ranging from brief sessions (52, 54, 74) to months, semesters, or year-long curriculum integration (39, 41, 75, 76). Brief-duration interventions, including those delivered over a few hours or days, reported positive immediate outcomes such as gains in knowledge, health literacy, critical literacy, and communicative literacy; although evidence regarding the sustainability of these effects over the longer term remains limited. Programs embedded within existing curricula or implemented over extended periods appeared more likely to influence broader competencies and organizational culture with sustained benefits (51). Interactive and peer-led strategies were associated with enhanced engagement and reduced stigma (45, 46, 52). Future research may explore this further, as these approaches may resonate more with the youth. Many studies relied on short follow-up periods, and context-specific tools, which may limit generalizability. The short duration of many interventions also makes it difficult to determine whether improvements in health literacy are sustained over time or translate into lasting behavioral change. This suggests a need for longer-term and recurrent school-based interventions that are integrated into curricula activities to better support sustained health literacy development across micro-, meso-, and macro-level contexts.

### 4.5 Implications for policy, practice, and future direction

The findings of this scoping review indicate that interventions implemented at the micro-, meso-, or macro-level were each associated with improvements in health literacy within the educational organization. No comparisons between low and high-socioeconomic position populations were evaluated in the included studies; therefore, no causal or differential effects on health equity can be inferred from the available evidence. Nevertheless, integrating health literacy into school curricula, teacher training institutions, and school accreditation processes may enhance sustainability. The benefits of advancing health literacy are profound; hence, at the organizational level, schools should adopt a whole-school approach that includes embedding health literacy into curricula across disciplines. This may possibly address inconsistencies noted in studies where health education (via taught subjects) is not statutory across the national curriculum (58). Furthermore, engaging families and communities may reinforce health literacy beyond school settings and may yield cumulative benefits across academic performance, well-being, and long-term population health outcomes.

There was a paucity of longitudinal research, limiting the capacity to capture the long-term impact of health literacy strategies. Future research should prioritize longitudinal designs and mixed-methods approaches to capture both quantitative and qualitative outcomes within the school setting. Teachers’ role is key, as advancing their health literacy has an indirect benefit and will ultimately lead to improvement in the students’ health literacy. Strengthening teachers’ mental health literacy will ultimately improve students’ support systems (32, 35). Therefore, it is vital to strengthen staff capacity through professional development. Policymakers in the educational sector should incorporate health literacy competencies into teacher education curricula and provide continuous professional development.

While policy creates enabling structures, implementation occurs at the organizational level. Therefore, schools should adopt a whole-school approach to implement health literacy policies. Whole-school approaches observed in peer education led to a cultural shift that influenced psychological and physical well-being, demonstrating the possibility of change within the school environment (46). Through this cultural shift, stigma about mental health dissipated, thus normalizing conversation about mental health support. Educational institutions should aim to embed health literacy across multiple subjects, including science, physical education, and social studies.

Although this review included studies from diverse regions, there was limited research in low- and middle-income countries. Thus, in terms of the geographic distribution of included studies in relation to global health literacy, the majority of studies were conducted in high-income countries, which may reflect established research infrastructure and policy prioritization in these settings. In contrast, the relative absence of studies from low- and middle-income regions highlights ongoing disparities in research capacity and investment. This highlights vital equity considerations, as unequal access to health literacy interventions and resources may further exacerbate existing health and educational disparities among students in low- and middle-income countries. Future research should prioritize and enhance health literacy research in low- and middle-income countries to support contextually relevant and equitable advancement of health literacy. Cross-national or country-comparative studies could also be explored, as this may identify contextually transferable best practices.

With increasing exposure to online health information and misinformation, future research should also expand digital health literacy and strategies to specifically combat health misinformation among students.

### 4.6 Strengths and limitations of the review

This scoping review provides a comprehensive mapping of recent literature on health literacy strategies adopted within educational settings. By drawing from multiple databases across health and education disciplines, the review captures interdisciplinary perspectives on health literacy strategies.

Limiting studies to peer-reviewed English-language studies may have excluded relevant research from non-English-speaking regions. Though the search included five databases, searching other bibliographic databases may have yielded additional published articles. Also, as this is a scoping review, the study did not conduct a formal quality appraisal or evaluation of risk of bias for the included studies, which precludes definitive conclusions regarding the effectiveness of the interventions. Hence, conclusions regarding effectiveness should be interpreted cautiously.

Despite these limitations, this scoping review offers insight into the strategies that can be adopted to advance health literacy in the educational sector to promote equity. Additionally, the breadth of included studies offers valuable insights into trends, gaps, and future directions.

## 5 Conclusion

Advancing health literacy within educational settings may represent a promising strategy for promoting health equity. Schools and universities are not merely sites of knowledge transmission but critical social institutions that shape lifelong health behaviors. Improved health literacy will not only promote health equities but also be an asset that leads to greater opportunities for health and lifelong learning (77). The evidence synthesized in this review suggests that when educational organizations intentionally integrate health literacy into their structures and curricula, measurable improvements in knowledge, self-efficacy, and well-being occur, which not only benefit students but also teachers and the whole community. The strongest and most equitable impacts may be observed when health literacy is embedded within organizational structures, educator training, and school environments.

Governments and policymakers in the educational sector may integrate health literacy into broader public health strategies, and mental health action plans to promote equity-focused implementation, which are essential to transforming the educational institutions into fully health-literate organizations capable of reducing health disparities. To achieve sustainable and equitable outcomes, future efforts may move beyond isolated “micro-level’ interventions toward systemic, policy-driven approaches that embed health literacy as a core function of educational institutions. Optimizing health literacy would act as an indirect enabler, strengthening current and future generations through ongoing significant national education reforms and their introduction in the school curriculum (78). Thus, positioning the educational sector as leaders in promoting health equity and improving students’ health trajectories.

## Data Availability

The original contributions presented in the study are included in the article/supplementary material, further inquiries can be directed to the corresponding author.
